# Evolution of the average avalanche shape with the universality class

**DOI:** 10.1038/ncomms3927

**Published:** 2013-12-19

**Authors:** Lasse Laurson, Xavier Illa, Stéphane Santucci, Ken Tore Tallakstad, Knut Jørgen Måløy, Mikko J Alava

**Affiliations:** 1 COMP Centre of Excellence, Department of Applied Physics, P.O. Box 11100, FI-00076 Aalto, Espoo, Finland; 2 Facultat de Física, Departament Estructura i Constituents de la Materia, Universitat de Barcelona, Martí i Franquès 1, 08028 Barcelona, Catalonia, Spain; 3 Laboratoire de physique, CNRS UMR 5672, Ecole Normale Supérieure de Lyon, 46 Allée d’Italie, 69364 Lyon Cedex 07, France; 4 Department of Physics, University of Oslo, PB 1048 Blindern, NO-0316 Oslo, Norway

## Abstract

A multitude of systems ranging from the Barkhausen effect in ferromagnetic materials to plastic deformation and earthquakes respond to slow external driving by exhibiting intermittent, scale-free avalanche dynamics or crackling noise. The avalanches are power-law distributed in size, and have a typical average shape: these are the two most important signatures of avalanching systems. Here we show how the average avalanche shape evolves with the universality class of the avalanche dynamics by employing a combination of scaling theory, extensive numerical simulations and data from crack propagation experiments. It follows a simple scaling form parameterized by two numbers, the scaling exponent relating the average avalanche size to its duration and a parameter characterizing the temporal asymmetry of the avalanches. The latter reflects a broken time-reversal symmetry in the avalanche dynamics, emerging from the local nature of the interaction kernel mediating the avalanche dynamics.

## Abstract (web summary)

The processes of earthquakes and plastic deformation are examples of systems which respond to slow external forces by avalanche dynamics. Here, the authors show how a fundamental fingerprint of such dynamics - the average shape of the avalanches - evolves with its universality class.

## Graphical abstract

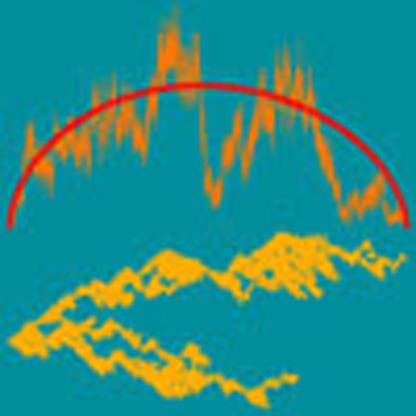

The theoretical interpretation of crackling noise^1^, observed in numerous systems, including the Barkhausen effect in ferromagnetic materials^2^,^3^, plastic deformation^4^,^5^,^6^, structural transitions^7^ and fracture^8^,^9^ of solids and earthquakes^10^, has found a formulation in terms of non-equilibrium phase transitions^11^. These transitions separate quiescent and active phases of the system, and naturally give rise to critical scaling^12^. In the vicinity of such a phase transition, the time evolution of the activity signal *V*(*t*) or the order parameter of the transition (for example, the interface velocity for a depinning transition) exhibits scale-free bursts or avalanches. Statistical analysis of such fluctuations, together with renormalization group calculations^13^, suggest that in general systems with avalanche dynamics can be classified into universality classes characterized by the values of the critical exponents, depending on, for example, the spatial dimension and the interaction range of the system.

The average temporal shape of bursts in a crackling noise signal is a fundamental signature of avalanches, and has been estimated for systems as diverse as plastically deforming crystals^14^, earthquakes^15^ and Barkhausen noise^16^,^17^,^18^. For the latter, the symmetric average avalanche shape observed in ferromagnetic films of intermediate thickness where the long-range dipolar interactions render the avalanche dynamics mean field-like has been explained within the ABBM model^19^, and shown to be given by an inverted parabola^16^,^20^,^21^,^22^. In thick enough samples eddy currents induce an effective mass for the propagating domain walls, visible as an asymmetry in the (mean-field-like) average shape of the Barkhausen pulses^17^. In general, clear-cut shape determinations should give strong indications of the underlying physics, such as the kind and range of interactions governing the avalanche dynamics.

Here we present a general scaling form for the average avalanche shapes for non-mean-field systems. It is verified within a large-scale numerical study of avalanches at the depinning transition of driven elastic interfaces in random media. We vary systematically the range of the elastic interaction kernel, and thus the universality class of the avalanche dynamics^23^, showing how the avalanche shape depends on the universality class. The average shape is to a high precision given by a function parameterized by the scaling exponent *γ* characterizing the scaling of the average avalanche size as a function of the avalanche duration, and a parameter *a* describing the temporal asymmetry of the average avalanche shapes. We find that an inherent asymmetry in the average avalanche shapes is present in systems where the interaction kernel is not fully non-local, reflecting the underlying broken time-reversal symmetry of the avalanche dynamics. Finally, we compare these results with experiments of planar crack front propagation, finding good agreement with the predictions of the scaling theory and the relevant depinning model.

## Results

### Average avalanche shape scaling function

To obtain a general scaling form for the average shape of the bursts in *V*(*t*) corresponding to avalanches of a given duration *T*, ‹*V*(*t* | *T*)〉, we start from the well-known result that the average avalanche size ‹*s*(*T*)〉 follows in the scaling regime


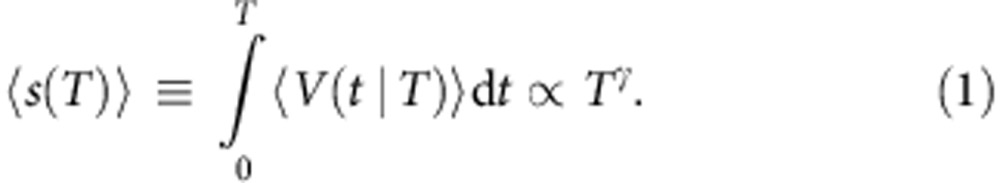


Thus, the amplitude of the average avalanche shape of avalanches of duration *T* has with *T* the relation ‹*V*(*t* | *T*)〉∝*T*^*γ*−1^, and is in general given by the form


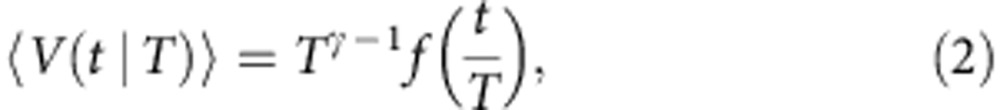


where *f(x)* is a scaling function characterizing the average temporal avalanche shape. We make the Ansatz that the average early-time growth of an avalanche is given by a power law of time, that is, ‹*V*(*t* | *T*)〉∝*t*^*δ*^ for 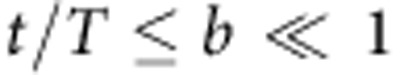. Given that equation (2) implies that ‹*V*(*t* | *T*)〉∝*T*^*γ*−1^ for any fixed *t*/*T*, it follows in particular that ‹*V*(*bT* | *T*)〉∝*T*^*γ*−1^. Therefore, in order for the postulated power law early-time growth to be compatible with this, one needs to have ‹*V*(*bT* | *T*)〉∝(*bT*)^*δ*^∝*T*^*γ*−1^, that is, *δ*=*γ*−1. Thus, the average early-time growth scales with *t* as


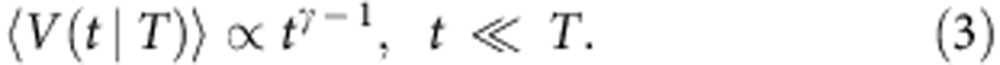


To arrive at an expression for ‹*V*(*t* | *T*)〉 satisfying equations (2) and (3), we multiply equation (3) by (1−*t*/*T*)^*γ*−1^, to describe the (symmetric) deceleration at the end of an avalanche, and obtain


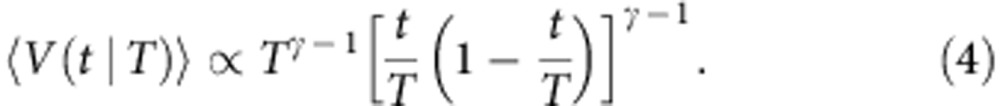


In mean-field systems, ‹*V*(*t* | *T*)〉 is known to be given in the scaling regime by the inverted parabola, predicted within the ABBM model in the limit of vanishing drive rate and demagnetizing factor, ‹*V*(*t* | *T*)〉∝*t*(1−*t*/*T*)≡*T*(*t*/*T*)(1−*t*/*T*) (ref. 16). This is in agreement with equation (4), given the mean-field value *γ*=2. Notice also that random walk bridges obey similar scaling with *γ*=3/2 (refs 24, 25).

**Figure 1:**
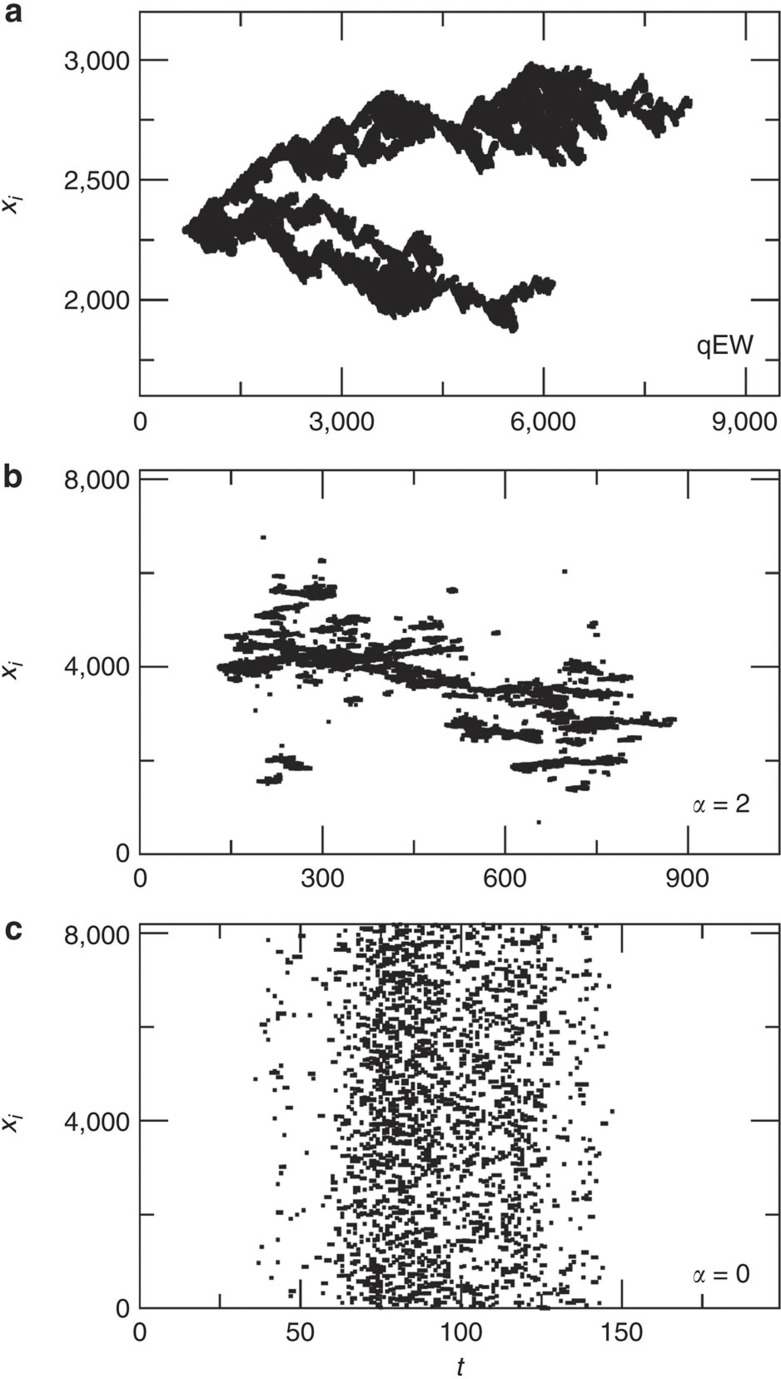
Space-time activity plots reveal broken time-reversal symmetry in avalanche dynamics. Examples of space-time activity of typical large avalanches for three cases: (**a**) the qEW equation with local elasticity, (**b**) the crack line model with non-local elasticity (*α*=2) and (**c**) the mean-field infinite range model. Notice the clear time-irreversible nature of the spatio-temporal avalanche structure in (**a**), and the reversible character in (**c**).

Although avalanches in mean-field systems have a symmetric average shape^16^, there is no fundamental reason why this should be true in general. Indeed, it is easy to see from the space-time activity plots of avalanches from, for example, the local quenched Edwards–Wilkinson (qEW) equation^26^ used in this study (see Fig. 1a) that the internal dynamics of the avalanches appears to violate time-reversal symmetry: the branching exhibited by the activity pattern at all scales clearly defines a direction of time. This means that the system is not Markovian: a possible signature of that is a temporal asymmetry in the average avalanche shape ‹*V*(*t* | *T*)〉 (ref. 24). For an increasing range of the elastic interactions, the time-irreversible nature of the activity pattern becomes less evident (Fig. 1b) and naturally vanishes for the mean-field infinite range model (Fig. 1c; see below for the definitions of the various interface depinning models): the activity pattern becomes a cloud of points without any internal time-irreversible structure.

Therefore, to account for the time-irreversible avalanche dynamics, we need to allow for a small temporal asymmetry in the average avalanche shape. Thus, we multiply equation (4) by 1−*a*(*t*/*T*−1/2), that is, a first order correction term allowing for an asymmetry quantified by *a*, and obtain


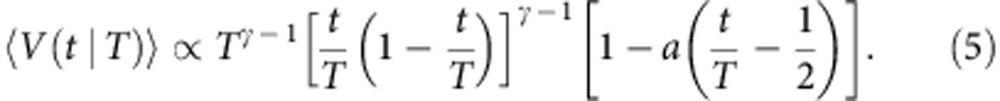


If *a*=0, equation (5) reduces to equation (4) and corresponds to a symmetrical avalanche shape. With *a*≠0, equation (5) describes a temporally asymmetric avalanche shape, with a positive or negative skewness for *a*>0 and *a*<0, respectively (see also Supplementary Note 1).

### Numerical simulations of interface depinning models

To check equation (5), we perform extensive simulations of a discretized model of a 1*d* elastic string or interface in a 2*d* random medium^23^, represented by a set of integer heights *h*_*i*_(*t*), *i*=1 … *L*, with *L* the system size. The lateral coordinates *x*_*i*_ of the interface are given by *x*_*i*_=*i*. The total force acting on the interface element *i* is


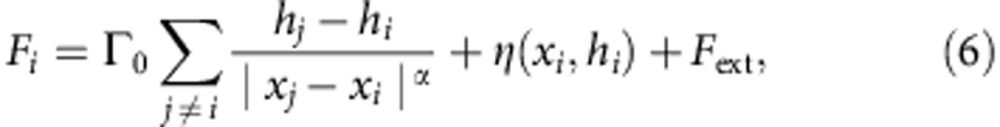


where the first term on the RHS represents the elastic interactions characterized by the exponent *α*, *η* is uncorrelated quenched disorder and *F*_ext_ is the external driving force. Notice that in the limit *α*→∞, the elastic interaction term becomes completely local, Γ_0_(*h*_*i*+1_+*h*_*i*__−__1_−2*h*_*i*_)≡Γ_0_∇^2^*h*_*i*_: thus, equation (6) reduces to the qEW equation. In the opposite limit (*α*→0) the system loses its spatial structure and we describe it by the mean-field infinite-range model, by replacing the elastic interactions in equation (6) by 
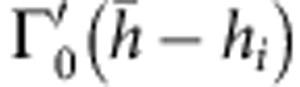
, with 
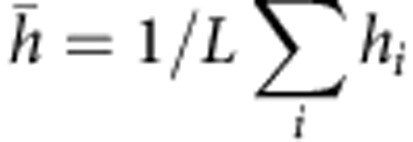
. For the intermediate case of *α*=2, equation (6) reduces to the long-range elastic string expected to describe, for example, planar crack fronts propagating along disordered weak planes between solid blocks^27^,^28^, contact lines of liquids spreading on solid surfaces^29^ and low-angle grain boundaries in plastically deforming crystals^30^. Furthermore, *α*≥3 belongs to the qEW class, whereas *α*=1 is expected to follow mean-field dynamics. The crackling noise signal is given by 
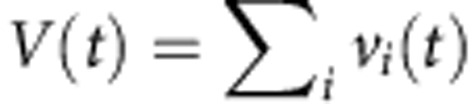
, where *v*_*i*_=*θ*(*F*_*i*_), with *θ* the Heaviside step function. For additional details, see Methods.

**Figure 2:**
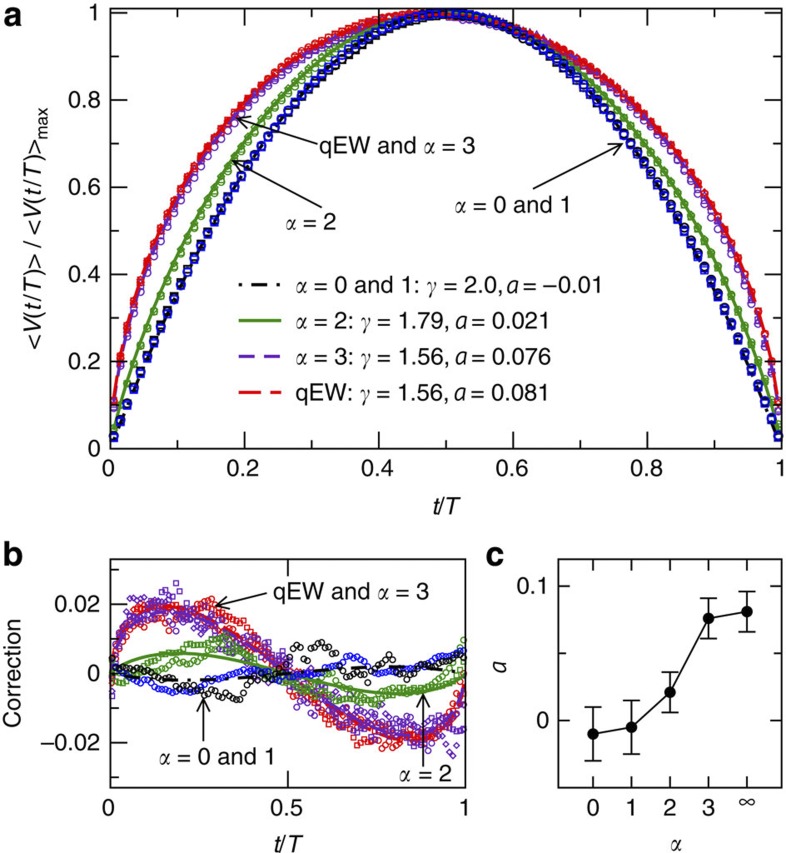
The average avalanche shapes from interface depinning models. (**a**) ‹*V*(*t*/*T*)〉/‹*V*(*t*/*T*)〉_max_ from the interface depinning simulations for different elastic kernels, ranging from the infinite range model (*α*=0, black symbols) to the local qEW equation (red symbols). The avalanche shapes corresponding to *α*=1, 2 and 3 are shown with blue, green and indigo symbols, respectively. Different symbols correspond to different duration ranges of the avalanches. Statistical error bars are negligible (not shown). The lines are fit to the data according to equation (5). (**b**) shows the asymmetry corrections to the average avalanche shapes, that is, ‹*V*(*t*/*T*)〉/‹*V*(*t*/*T*)〉_max_−[4(*t*/*T*)(1−*t*/*T*)]^*γ*−1^. Scatter of the data points corresponds to the magnitude of the statistical fluctuations. The asymmetric parts of equation (5) are shown with lines, with the best-fit parameters given in the legend. (**c**) shows the evolution of the asymmetry parameter *a* with *α*. The error bars are estimated from the scatter (s.d.) of the best-fit *a*-values obtained for different duration ranges within the scaling regime.

As expected, ‹*s*(*T*)〉 scales with *T* according to equation (1) (see Supplementary Fig. S1 and Supplementary Note 2). We find three different values of *γ*, that is, *γ*=2.0±0.01 for *α*≤1, *γ*=1.79±0.01 for *α*=2 and *γ*=1.56±0.01 for *α*≥3. These values are in agreement with earlier results, either directly or via scaling relations^12^,^16^,^27^,^31^. Fitting equation (5) to the ‹*V*(*t* | *T*)〉 data for various *α* and different ranges of *T* in the scaling regime reproduces well these *γ*-exponents (Fig. 2a,b; see also Supplementary Figs S2–S5 and Supplementary Notes 3 and 4). The avalanche shapes exhibit an asymmetry, with the *a*-parameter evolving continuously from −0.01±0.02 (for the infinite range model) to 0.081±0.015 (for the qEW equation), as the interaction range is reduced (Fig. 2c; see also Supplementary Fig. S6 and Supplementary Note 5). The corresponding skewness (computed by interpreting ‹*V*(*t* | *T*)〉 as a probability density^17^) of the avalanches exhibits a similar evolution with *α* (Supplementary Fig. S7, Supplementary Note 6). Thus, avalanches whose dynamics is governed by interaction kernels that are not fully non-local are temporally asymmetric, as illustrated by the time-irreversible nature of the corresponding space-time activity patterns (Fig. 1).

### Planar crack front propagation experiments

**Figure 3:**
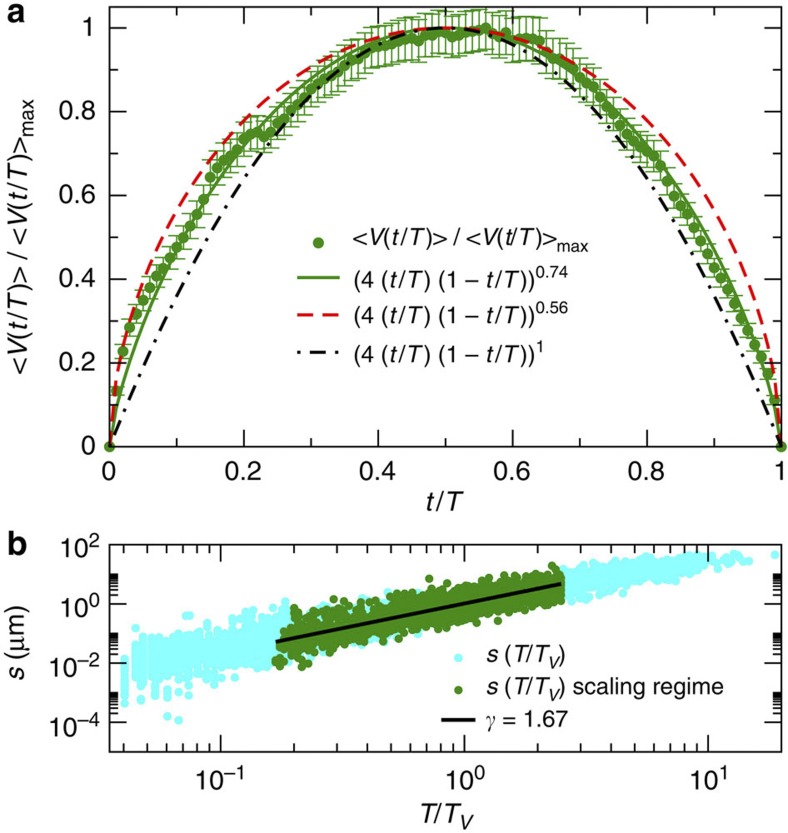
The average avalanche shape in planar crack front propagation experiments. (**a**) Symbols with error bars (corresponding to statistical error (s.e.m.), quantified by the s.d. of *V*(*t*/*T*) normalized by the square root of the number of avalanches) show the measured average avalanche shape, along with a best fit of equation (4), or equation (5) with *a*=0, corresponding to *γ*=1.74±0.08. The mean-field (dash-dotted black line) and the symmetrized qEW (dashed red line) results are shown for comparison. (**b**) shows the scaling of the avalanche size *s* with the normalized duration *T*/*T*_*V*_ (see Methods), with a fit to the scaling regime giving *γ*=1.67±0.15.

Finally, we consider data from planar crack front propagation experiments^9^,^32^, as an example of an experimental system with non-mean-field avalanche dynamics, see Methods for details. The scaling of the average size of the avalanches of crack front propagation as a function of their duration is shown in Fig. 3b. In the scaling regime, these are characterized by *γ*=1.67±0.15, in agreement with the 1*d* non-local elasticity depinning model with *α*=2 (refs 27, 28), see also Supplementary Fig. S8. The average avalanche shape is shown in Fig. 3a. Owing to the non-negligible statistical fluctuations present in the data, it is not possible to detect the small asymmetry predicted by the crack line model: thus, we choose to fit the leading-order behaviour, equation (4), to the data (see also Supplementary Fig. S9). This leads to *γ*=1.74±0.08, in agreement with the *γ*-value obtained from the fit to the shape obtained from the crack line model^27^,^28^. Notice also that the experimental shape clearly differs from both the mean-field inverted parabola and the shape expected for the local qEW equation.

## Discussion

We have shown how the average avalanche shape of systems exhibiting crackling noise depends on the universality class of the avalanche dynamics. It is a fundamental fingerprint of an avalanching system and extrapolates when tuning elastic interactions between an inverted parabola for mean-field systems and a shape close to a semicircle for the 1*d* short-range interface. The broken time-reversal symmetry in the avalanche dynamics emerging from the spatially localized interactions is manifested as a temporal asymmetry in the avalanche shape evolving with the interaction range (see also Supplementary Discussion). Thus, such asymmetries should be looked for in experimental data in systems where the interactions mediating the avalanche dynamics are not fully non-local. These include, for example, domain wall dynamics in magnetic thin films^33^ and fluid invasion into disordered media^34^,^35^.

## Methods

### Numerical simulations of interface depinning models

**Figure 4:**
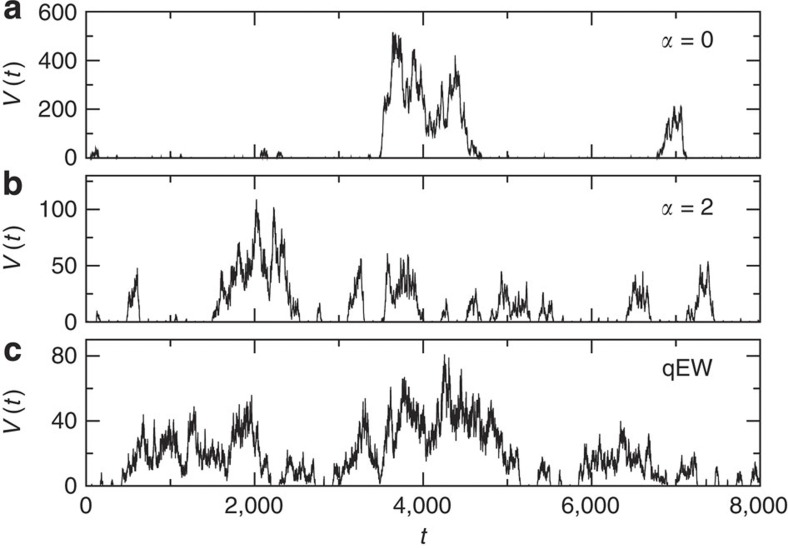
Examples of the crackling noise or interface velocity signals from the interface depinning models. The interface velocity 
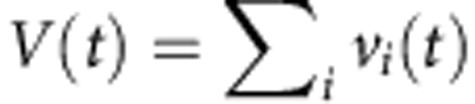
 for different ranges of the elastic interactions, with (**a**) the infinite range model, (**b**) the crack line model (*α*=2) and (**c**) the local qEW equation shown here. Owing to the quasistatic driving mechanism, the avalanches can be unambiguously defined without thresholding. Here, for visual clarity a fixed quiet time *t*_q_=100 has been added between each avalanche. All three signals are of the same length.

We simulate the interface depinning model, equation (6) with periodic boundary conditions. The parallel dynamics of the interface is defined in discrete time *t* by setting the local velocity *v*_*i*_(*t*)≡*h*_*i*_(*t*+1)−*h*_*i*_(*t*)=*θ*(*F*_*i*_), where *θ* is the Heaviside step function. The interface is driven with a quasistatic constant velocity drive, where avalanches are triggered by increasing *F*_ext_ just enough to make exactly one interface element unstable (that is, *F*_*i*_>0 for some *i*) whenever the previous avalanche has ended. Thus, avalanches can be defined unambiguously without thresholding^28^. During an avalanche, *F*_ext_ is decreased at a rate proportional to the instantaneous avalanche velocity, 
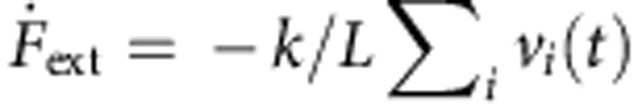
. *k* is a parameter analogous to the demagnetizing factor for ferromagnetic domain walls^36^ or to the elastic stiffness of the specimen-machine system in a mechanical loading experiment^37^ and controls the cut-off of the avalanche distribution, with the cut-off size *s*_0_ obeying 
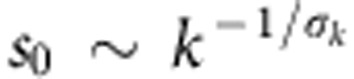
. The crackling noise signal of interest is given by 
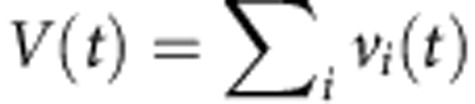
. To compute the average avalanche shapes, we collect a large ensemble of avalanches from various duration ranges. For *T* in the scaling regime, the average shapes corresponding to the various duration ranges fall onto a single curve after normalizing with the maximum amplitude, ‹*V*(*t* | *T*)〉_max_. The simulations are performed in large system sizes (*L*=8,192=2^13^ for *α*≥3, *L*=32,768=2^15^ for *α*=2, *L*=131,078=2^17^ for *α*=1 and *L*=8,388,608=2^23^ for the infinite range model), and by using sufficiently small *k*-values such that the avalanche cut-off 
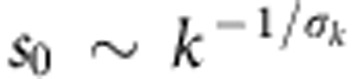
 is large. Examples of the crackling noise signals 
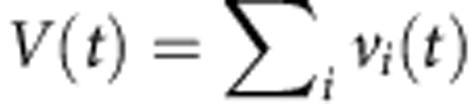
 obtained from the model for different interaction ranges are shown in Fig. 4.

### Planar crack front propagation experiments

**Figure 5:**
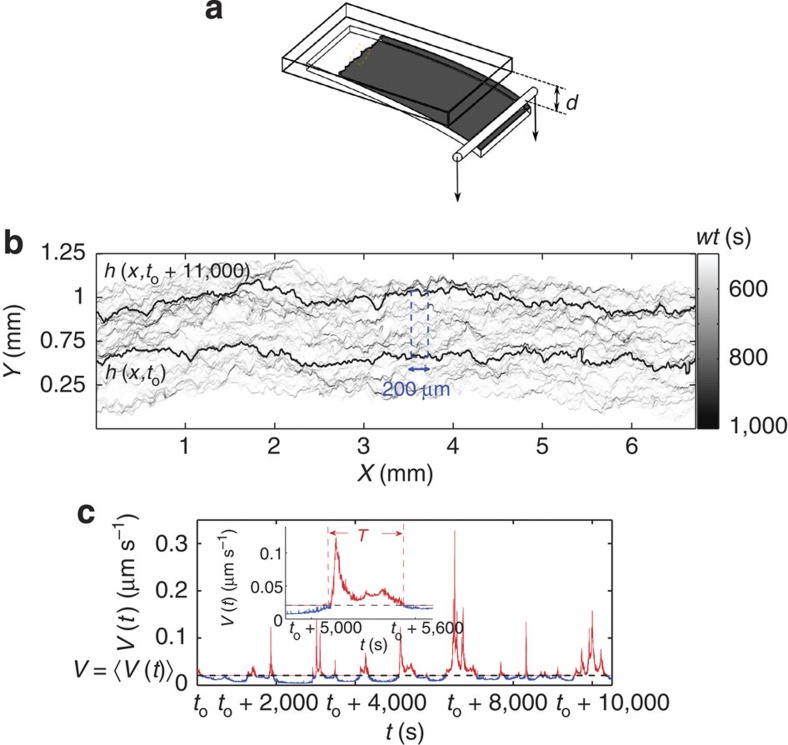
The experimental setup. (**a**) Creep experiments of planar crack propagation are performed by imposing a constant normal displacement *d* to the bottom plate while keeping the upper plate fixed. (**b**) By observing a small central region with a camera, the local waiting times *wt* of the crack front are measured at each location, with dark (light) regions corresponding to long (short) waiting times, respectively. The two solid black lines correspond to two examples of the instantaneous crack front profile. (**c**) The velocity signal *V*(*t*) is measured at the scale of 200 μm (dashed blue line in (**b**)), by considering the average of the local velocities *v*(*x*,*y*), defined as the inverse of the local waiting times. The inset shows a close-up of an avalanche of duration *T*, defined as a continuous occurrence of the global velocity *V*(*t*) above its average *V*=‹*V*(*t*)〉_*t*_.

To extract the average avalanche shape for the planar crack propagation experiments, slow creep motion of a planar crack front propagating along the heterogeneous weak plane of a transparent poly (methyl methacrylate) block, made of two sintered rough Plexiglas plates (of dimensions (27, 14 and 1) cm for the top and (30, 12 and 0.4) cm for the bottom plate, respectively) is studied^8^,^9^,^32^. We imposed a constant normal displacement *d* to the bottom plate while the upper one is fixed, resulting in a quasi-mode I creep growth of the crack, see Fig. 5a. The interfacial fracture front was observed in a small central region (to avoid boundary effects) corresponding to around 4.48 mm and 6.72 mm in the direction of propagation and along the front, respectively. The propagation of the crack front is monitored optically by using a camera with a high frame rate compared with the average crack front velocity, recording typically 20,000 images of 2,000 × 3,000 pixels with a pixel size of *r*=2.24 μm at a rate ranging from 1 to 60 fps. From these images, we define the local velocity *v* along the crack front by measuring the waiting time (*wt*) the front has spent at a given position (*x*,*y*) and setting *v*(*x*,*y*)=*r*/*wt*(*x*,*y*) (ref. 9), see Fig. 5b. We consider here the ‘global’ velocity of the crack front *V*(*t*), computed (that is, spatially averaged from the local velocities) at a length scale of *δx*=200 μm, larger than the correlation length of 100 μm of the local velocities^32^, leading to more than 30 different and independent crackling noise signals *V*(*t*) for each creep test (since the lateral size of the imaging area divided by *δx* is (6720 μm)/(200 μm)=33.6). Five different creep experiments with low average crack front velocities *V*=‹*V*(*t*)〉_*t*_ (*V* ranging from 0.017 μm s^−1^ to 1.13 μm s^−1^) are considered, resulting in more than 150 crackling noise signals containing in total more than 4,000 bursts or avalanches. An example of a signal *V*(*t*) is shown in Fig. 5c.

During a creep test, the average crack front velocity decreases slowly. However, the time intervals studied here are short enough to consider the average crack front velocity constant within such time windows. Avalanches are defined as a continuous occurrence of *V*(*t*) above its average value *V*=‹*V*(*t*)〉_*t*_. We have verified that by choosing another threshold values in a reasonable interval around *V* does not influence the extracted results. The average value is subtracted from *V*(*t*) to define the avalanche size (that is, the integral of *V*(*t*)−*V*) and shape. As the different experiments have a different average velocity, we consider the rescaled avalanche durations *T*′=*T*/*T*_*V*_, where the time scale *T*_*V*_ is given by *T*_*V*_=*r*/*V*. Also, very short and very long avalanches might affect the scaling behaviour as well as the avalanche shape, owing to a poor resolution and a lack of statistics, respectively. Therefore, we consider only avalanches that last longer than *T*=25 *δt*, (the temporal resolution *δt* varying between 1/60 s up to 1 s for the various experiments performed). In the inset of Supplementary Fig. S8, we show the probability density functions *P*(*T*′) of the normalized durations for all avalanches extracted and for such a subset, that is, *P*(*T*′|*T*>25). We can observe an exponential cut-off for large duration *T*′>2.5, which provides the upper limit for the scaling range we will consider, leading finally to the study of slightly more than 1,500 avalanches. A power-law fit to the avalanche size *s* as a function of the normalized durations *T*′ for those avalanches yields an exponent *γ*=1.67±0.15 (as shown in Fig. 3b, and in the Supplementary Fig. S8), in good agreement within error bars with the 1*d* depinning model with *α*=2 (ref. 27). We finally normalize the amplitudes of those avalanches by *T*′^*γ*−1^ and compute their average shape ‹*V*(*t* | *T*′)〉. The resulting average avalanche shape normalized by the maximum amplitude, ‹*V*(*t* | *T*′)〉/‹*V*(*t* | *T*′)〉_max_, is shown in Fig. 3a.

Finally, we discuss the effect of the limited statistics available from the experiments. In order to estimate the amount of statistics required to observe the predicted asymmetry, we estimate the statistical error bars *δ*_*N*_(*V*/*V*_max_) of the average avalanche shapes averaged over *N* avalanches as 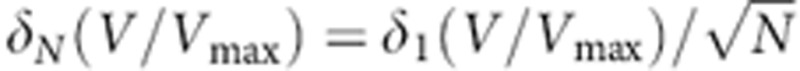, where *δ*_1_(*V*/*V*_max_)≈1 is the fluctuation magnitude of an individual avalanche. As the typical difference between the normalized avalanche shape of the depinning model with *α*=2 and the corresponding symmetrical shape is roughly 0.005 or less (see Fig. 2c), the error bars have to be smaller than that to be able to distinguish the asymmetry from the data. Setting *δ*_*N*_(*V*/*V*_max_)=0.005 corresponds to *N*=40,000, which should be interpreted as a lower limit. Thus, we estimate the order of magnitude of the number of avalanches *N* required to make conclusions regarding the asymmetry to be of the order of *N*=10^5^, that is, two orders of magnitude more than the number of avalanches we have at our disposal from the experiments. Indeed, this can also be seen from the data: in Supplementary Fig. S9 we show again the experimental average avalanche shape, along with a fit of equation (4). The inset displays their difference, showing clearly that the very small correction to the symmetric shape predicted by the crack line model is hidden by statistical noise.

## Author contributions

L.L. and X.I. designed and performed the numerical modelling. L.L. and M.J.A. developed the scaling theory. S.S., K.T.T. and K.J.M. performed and analysed the experiments. L.L. wrote the first draft of the manuscript. All authors contributed to improve the manuscript.

## Additional information

**How to cite this article:** Laurson, L. *et al.* Evolution of the average avalanche shape with the universality class. *Nat. Commun.* 4:2927 doi: 10.1038/ncomms3927 (2013).

## Supplementary Material

Supplementary Information Supplementary Figures S1-S9, Supplementary Notes 1-6, Supplementary Discussion and Supplementary References
