# Supplementary material for: Evolution of the average avalanche shape with the universality class
Source: Nat Commun. 2013 Dec 19;4:2927. doi: 10.1038/ncomms3927 (PMC3905775; doi:10.1038/ncomms3927)
Supplement: Supplementary Information — Supplementary Figures S1-S9, Supplementary Notes 1-6, Supplementary Discussion and Supplementary References [file ncomms3927-s1.pdf]

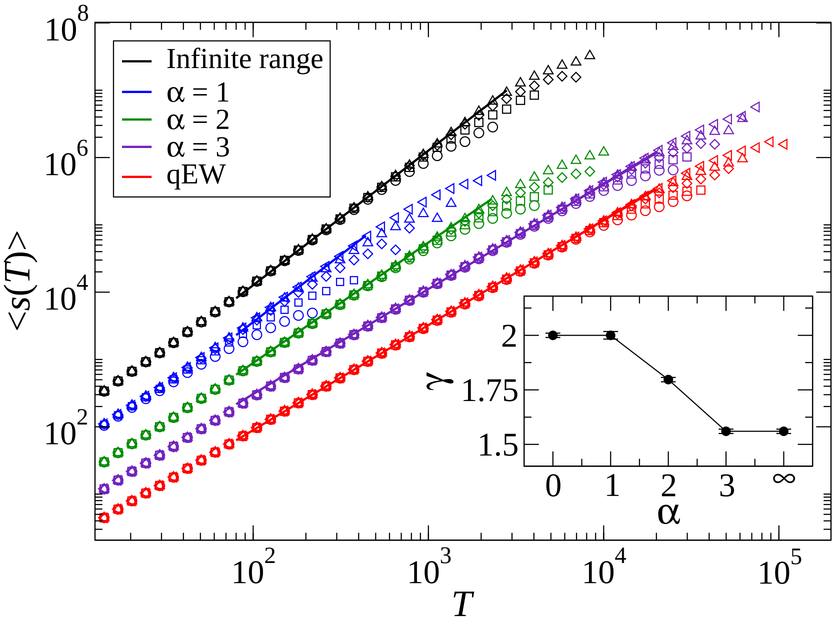


**Supplementary Figure** S1: Scaling of the avalanche size vs duration in the interface depinning models. The average avalanche size $\langle s(T) \rangle$ as a function of the duration $T$ for different $\alpha$, ranging from the infinite range model ($\alpha = 0$) to the local qEW equation. Different symbols correspond to different values of $k$, and the datasets for different $\alpha$ have been displaced vertically for clarity. The inset shows the $\gamma$-exponent as a function of $\alpha$ (with the qEW equation corresponding to $\alpha \to \infty$) estimated by fitting a power law to the data in the main figure. The error bars reflect the scatter of the $\gamma$-values (s.d.) obtained by different choices of the fitting interval.

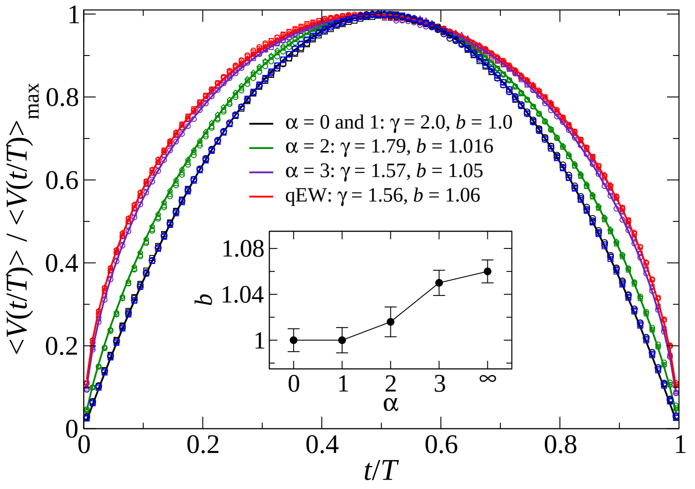


**Supplementary Figure** S2: Alternative fitting form to account for the temporal avalanche asymmetry. Main figure: The average avalanche shapes from the interface depinning model (symbols), with the solid lines corresponding to fits of equation (S3). The inset shows the best-fit value of the asymmetry parameter $b$ as a function of $\alpha$. The error bars are estimated from the scatter of the best-fit $b$-values (s.d.) obtained for different duration ranges within the scaling regime.

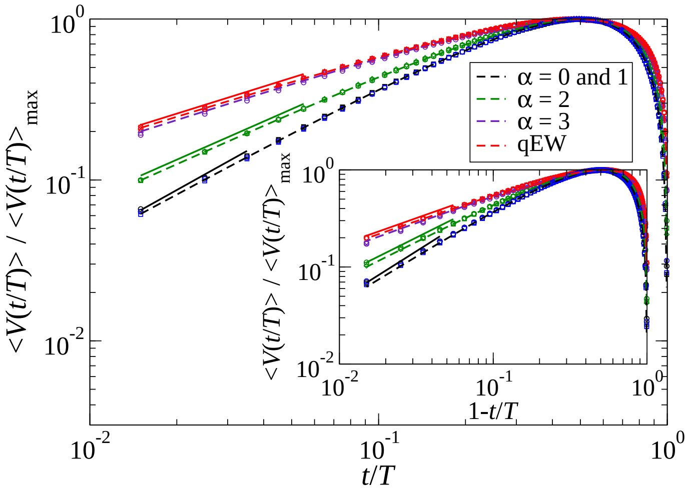


**Supplementary Figure** S3: Early-time growth and final deceleration of avalanches. Main figure: The average early-time growth of the avalanches for different interaction kernels, ranging from the mean field infinite range model (black symbols) to the local qEW model (red symbols). $\alpha = 1$, $\alpha = 2$ and $\alpha = 3$ are shown with blue, green and indigo symbols, respectively. Different symbols correspond to different avalanche duration ranges. The dashed lines correspond to fits of equation (5) of the main article, while the solid lines indicate the expected early time scaling, equation (S4), with $\gamma - 1 = 0.56$ (red line), 0.79 (green line) and 1.00 (black line), respectively. The fitting parameters are as in Fig. 2 of the main article. Inset: The same as above but for the late-time avalanche deceleration. The dashed lines correspond to equation (5) of the main article with the substitution $t/T \to 1 - t/T$, and the solid lines the expected late-time scaling, equation (S5), with the same values of $\gamma - 1$ as in the main figure.

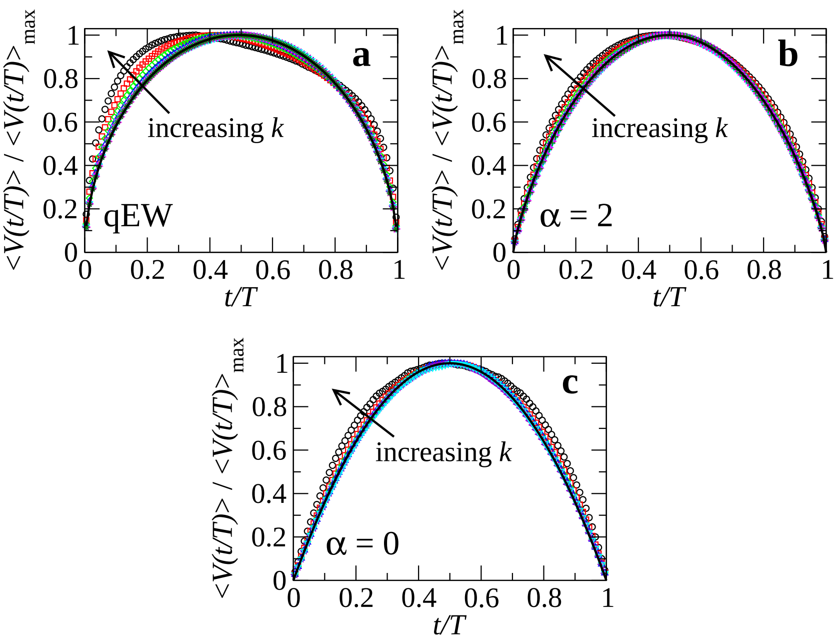


**Supplementary Figure** S4: Avalanche shapes get distorted in the cutoff region. Within the $k$-induced cutoff region of the avalanche distributions, the avalanche shapes are modified from their scaling regime shapes (black solid lines), and exhibit large positive skewness in the non-mean field cases. The mean field $\alpha = 0$ case exhibits symmetric flattening [16,20]. (**a**) qEW model, (**b**) $\alpha = 2$, (**c**) $\alpha = 0$.

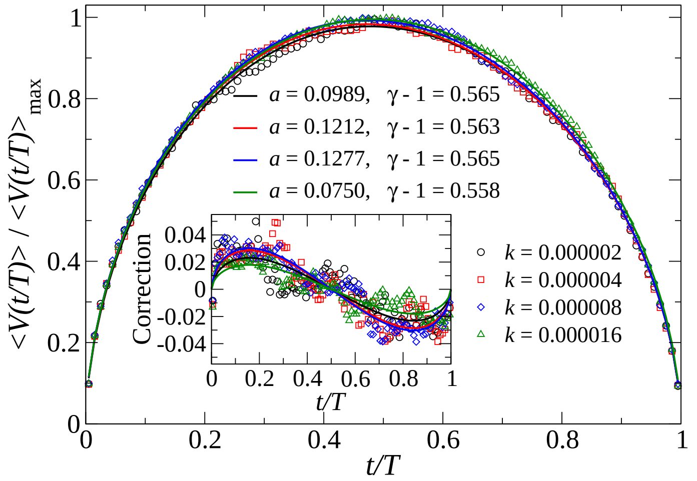


**Supplementary Figure** S5: Average avalanche shape in a continuous time model. Main figure: The average avalanche shapes in the scaling regime of the continuous version of the qEW equation (symbols). The solid lines correspond to fits of equation (5) of the main text to the data. The inset shows the asymmetry correction, i.e. the $\langle V(t/T)\rangle/\langle V(t/T)\rangle_{\max}$ data from which the symmetrical part of the Ansatz (equation (5) of the main text with $a = 0$) with best-fit values for $\gamma$ (indicated in the legend) has been subtracted (symbols), along with the best-fit asymmetry corrections of the form of equation (5) of the main text (solid lines). While the statistical fluctuations are larger than in the discrete version (main text), a similar asymmetry (with $a \approx 0.1$) is present also in this case, highlighting the robustness of the temporal asymmetry of the avalanches.

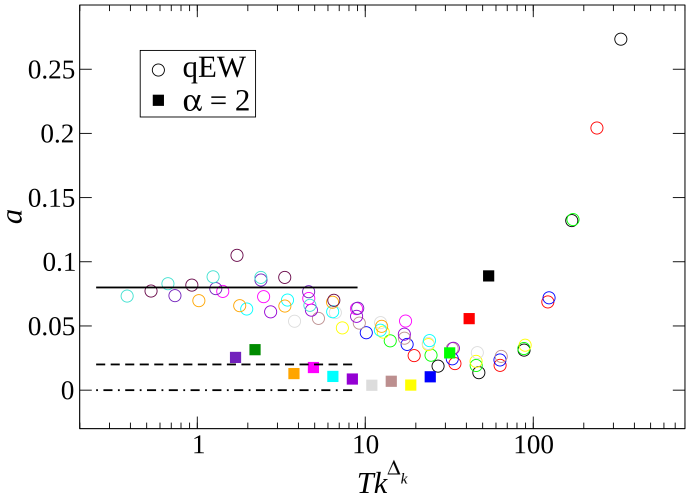


**Supplementary Figure** S6: Scaling of the temporal avalanche asymmetry. Scaling of the asymmetry parameter $a$ with the rescaled avalanche duration $Tk^{\Delta_k}$, where $\Delta_k$ is the scaling exponent characterizing the cutoff scaling of the avalanche duration distribution [see equation (S7)]. Open circles correspond to the qEW model (with $\Delta_k = 0.473$), while filled squares show the data for the crack line model, $\alpha = 2$ (with $\Delta_k = 0.385$). Different colors correspond to different $k$-values. The solid line shows the $a = 0.08$ (qEW scaling regime) case, while the dashed and dashed-dotted lines correspond to $a = 0.02$ ($\alpha = 2$ scaling regime) and $a = 0$ ($\alpha = 0$), respectively.

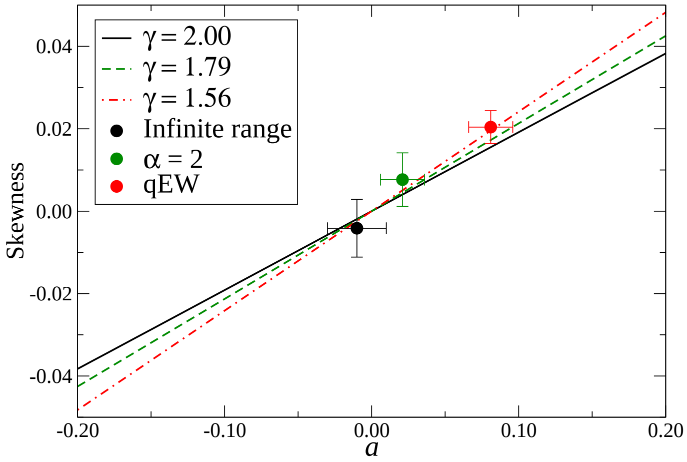


**Supplementary Figure** S7: Relation between the asymmetry parameter $a$ and the avalanche skewness. Lines show the numerically evaluated avalanche skewness corresponding to equation (S2), computed by using the definition of skewness given in equation (S8), as a function of the asymmetry parameter $a$ for three different values of $\gamma$. These $\gamma$-values correspond to the three universality classes of interface depinning observed when varying the interaction range by tuning $\alpha$. The symbols with errorbars correspond to the measured skewness-$a$ pairs in the interface depinning model for $\alpha = 0$, $\alpha = 2$, and for the qEW equation. The error bars are estimated from the scatter of the fitting parameter values (s.d.) obtained for different duration ranges within the scaling regime.

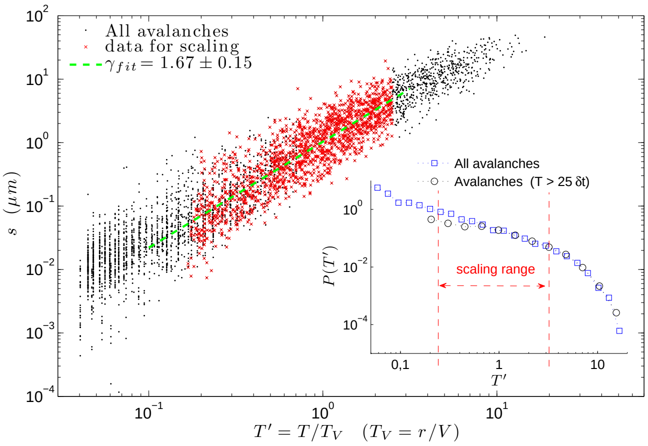


**Supplementary Figure** S8: Scaling of the avalanche sizes $s$ as function of the normalized durations $T'$. The inset shows the pdf's of the normalized durations $T'$ for all the avalanches extracted and the subset corresponding to avalanches 25 times longer than the time units $\delta t$, which varies between 1/60 s and 1s. The pdf's cut-off provides the upper limit of the scaling range studied. Within this range, a fit of the size $s$ versus durations $T'$ provide the scaling exponent $\gamma = 1.67 \pm 0.15$.

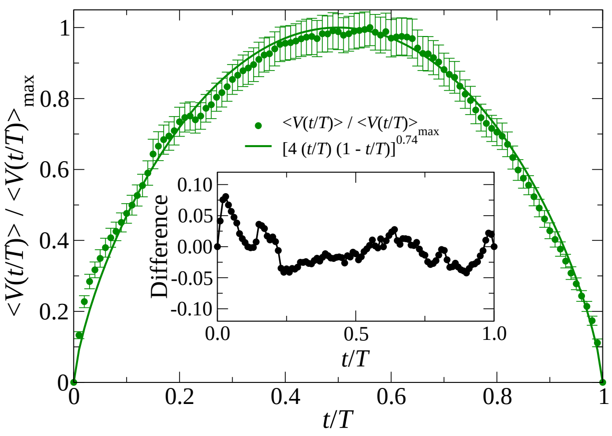


**Supplementary Figure** S9: An attempt to measure the avalanche shape asymmetry in crack propagation experiments. The main figure shows the average avalanche shape from the planar crack front propagation experiments (symbols), along with the fit of equation (S1) (solid line). The inset displays the difference between these two, showing that the very small asymmetry predicted by the crack line model cannot be observed due to statistical noise.

**Supplementary Note 1: Choice of the fitting form to characterize asymmetric avalanche shapes.** The scaling arguments presented in the main text lead to a symmetric average avalanche shape, given by

$$\langle V(t|T)\rangle \propto T^{\gamma-1}\left[\frac{t}{T}\left(1-\frac{t}{T}\right)\right]^{\gamma-1}. \tag{S1}$$

Due to the time-irreversible nature of the avalanche dynamics mediated by spatially localized elastic interaction kernels (see Fig. 1 of the main text), we need to generalize equation (S1) to include a possibility for a small temporal asymmetry. Since in finite-dimensional systems no exact solution of the problem is available, there is no theory that would tell us how equation (S1) should be modified, and obviously there is not a unique way of choosing such a modification. However, due to the smallness of the observed asymmetry, practically any correction describing a small asymmetry will work: All we need is a generalization of equation (S1) which includes a parameter that can be used to quantify the temporal asymmetry of the avalanches. Equation (5) of the main text, i.e.

$$\langle V(t|T)\rangle \propto T^{\gamma-1}\left[\frac{t}{T}\left(1-\frac{t}{T}\right)\right]^{\gamma-1}\left[1-a\left(\frac{t}{T}-\frac{1}{2}\right)\right], \tag{S2}$$

with $a > 0$ and $a < 0$ corresponding to positive and negative skewness, respectively, was chosen simply because it is the simplest form including a possibility for a small asymmetry which is of the form of a leading order shape (equation (S1)) plus a first order (linear) correction quantified by a single parameter $a$. It can be motivated also by the fact that a recent first order $\epsilon$-expansion around the upper critical dimension $d_c$ leads to a result of essentially this form [38]. Higher order terms could be included, e.g. by fitting a complete set of polynomials [18], but due to the smallness of the observed asymmetries, the lowest order correction to the symmetric shape is sufficient to fully capture the average avalanche shapes.

Moreover, since the sole purpose of the asymmetry correction here is to provide a fitting form allowing to quantify the asymmetry (via the value of $a$, which is obviously also related to the skewness of the avalanches, see Supplementary Note 6), also other forms of the asymmetry corrections would be equally suitable. An example thereof would be given by

$$\langle V(t|T)\rangle \propto T^{\gamma-1}\left[\frac{t}{T}\left(1-\frac{t}{T}\right)^{b}\right]^{\gamma-1}, \tag{S3}$$

where $b = 1$ describes a symmetric avalanche shape, and $b > 1$ and $b < 1$ correspond to positive and negative skewness, respectively. Supplementary Figure S2 shows the same data as in Fig. 2 of the main article, with fits of equation (S3) shown as solid lines. The best-fit value of the asymmetry parameter $b$ exhibits a similar evolution with $\alpha$ as the $a$-parameter in Fig. 2 of the main text, apart from the shift from $a$-values close to 0 to $b$-values close to 1.

**Supplementary Note 2: The scaling of the average avalanche size with duration.** In Supplementary Figure S1, we show the scaling of the average avalanche size $\langle s(T)\rangle = \int_0^T \langle V(t|T)\rangle dt$ with the duration $T$ for the various $\alpha$-values. In the scaling regime, these obey scaling of the form $\langle s(T)\rangle \sim T^{\gamma}$, with $\gamma = 1.56 \pm 0.01$ for $\alpha \geq 3$, $\gamma = 1.79 \pm 0.01$ for $\alpha = 2$, and $\gamma = 2.00 \pm 0.01$ for $\alpha \leq 1$. These values are in excellent agreement with earlier results, either directly or via scaling relations [12,16,27,31]. In particular, they are reproduced remarkably well by fitting the scaling form, equation (5) of the main text, to the average shapes of the avalanches, $\langle V(t|T)\rangle$, for different values of $\alpha$.

**Supplementary Note 3: The early-time avalanche growth and deceleration at the end of an avalanche.** The scaling form for the average avalanche shape, equation (5) of the main text, is based on the idea that the early and late time scaling of the avalanche shapes should obey

$$\langle V(t|T)\rangle \propto t^{\gamma-1}, \quad t \ll T, \tag{S4}$$

and

$$\langle V(t|T)\rangle \propto (1-t/T)^{\gamma-1}, \quad t \to T, \tag{S5}$$

respectively. The asymmetry correction in equation (5) of the main text does not modify the leading order scaling of equations (S4) and (S5). Thus, we can numerically verify the scaling arguments that lead to equation (5) of the main text, by directly looking at the power law scaling of the early and late time average avalanche shapes. In Supplementary Figure S3, we show the early (main figure) and late (inset) time scaling of the avalanche shapes for different $\alpha$, along with fits of equation (5) of the main text (dashed lines). The solid lines correspond to the expected pure power law scaling corresponding to equations (S4) and (S5), demonstrating clearly that the assumptions behind the scaling arguments which lead to equation (5) of the main text are valid. Note that beyond a certain range in time, one expects the general shape function to take over leading to a deviation from the power-law.

**Supplementary Note 4: Continuous time simulations of the interface depinning model.** To check that the avalanche shapes are not affected by the discretized dynamics of the interface depinning model, we also consider the continuous time version of the qEW equation with the local instantaneous velocity proportional to the total force acting on the interface element $i$,

$$\frac{\partial h_i}{\partial t} = \Gamma_0 \nabla^2 h_i + \eta(i, h_i) + F_{ext}. \tag{S6}$$

In equation (S6), $h_i$ and $t$ are continuous variables. To model $\eta$ as a continuous short-range correlated random force, we generate for each $i$ a cubic spline passing through regularly spaced uncorrelated Gaussian random points. A similar quasistatic constant velocity drive as in the discrete case is used, with $\dot{F}_{ext} = -k/L \sum_i v_i(t)$ during the avalanches (defined as periods of time when the average interface velocity $V(t) \equiv 1/L \sum_i v_i(t)$ exceeds a small threshold value $V_{th}$), and $F_{ext}$ is slowly ramped up in between avalanches (i.e. when $V < V_{th}$) until the next avalanche is triggered (i.e. when $V$ again exceeds $V_{th}$).

Equation (S6) is integrated numerically with a system size $L = 2048$, to collect a large ensemble of avalanches. While the statistics obtained with such a continuous time simulation is by necessity not as good as in the discrete case, we are still able to observe that the scaling regime average avalanche shape $\langle V(t,T) \rangle$ is in agreement with that obtained from the discrete qEW equation. Supplementary Figure S5 shows the average shapes from a fixed duration range for different values of $k$, which again controls the avalanche cutoff and thus the extension of the scaling regime. By fitting equation (5) of the main text to the data, we observe that while the scatter in the fitting parameters is obviously larger due to lack of statistics, both the $\gamma$-exponent and the asymmetry parameter $a$ are in agreement with those obtained for the discrete model. Also the shape of the asymmetry correction shown in the inset of Supplementary Figure S5 is very similar to that observed for the discrete qEW equation (Fig. 2b of the main text). This indicates in particular that the asymmetry we observe in the avalanche shapes is not an artifact of the discretization used in simulating the depinning model.

**Supplementary Note 5: Scaling of the temporal avalanche asymmetry.** Our results show that in the scaling regime, spatially localized interactions lead to a temporally asymmetric average avalanche shape. The $a$-values characterizing the asymmetry are found to be independent of $T$ and $k$ as long as the condition of the scaling regime, i.e. $1 \ll T \ll T_0 \propto k^{-\Delta_k}$, where $\Delta_k$ is a scaling exponent characterizing the dependence of the cutoff scale of the duration distribution of the avalanches on $k$, is satisfied. In the cutoff regime, $T \sim T_0$, the avalanche shapes get distorted, see Supplementary Figure S4. As can be observed in Supplementary Figure S4, this distortion is also manifested as an increase in the avalanche asymmetry/skewness in the non-mean field cases. In general the best-fit asymmetry parameter $a$ follows a simple scaling form,

$$a(T, k) = f(Tk^{\Delta_k}). \tag{S7}$$

In Supplementary Figure S6, we show data collapses of the best-fit $a$ according to equation (S7). These are obtained by using literature values for the scaling exponents, and a scaling relation $\Delta_k = z/(\mu + d)$ [2], where $z$ is the dynamic exponent ($z = 1.42$ [39] and $z = 0.770$ [31] for the qEW and crack line models, respectively) and $\mu = 2$ for the local qEW model and 1 for the non-local crack line model with $\alpha = 2$. This results in $\Delta_k = 0.473$ and 0.385 for the qEW model and the $\alpha = 2$ case, respectively. In Supplementary Figure S6, one can clearly observe a different constant non-zero value of $a$ for small $Tk^{\Delta_k}$ (i.e. the scaling regime) for each of the two non-mean field cases, whereas for larger $Tk^{\Delta_k}$ the cutoff mechanism (here the "demagnetizing field" $k$) distorts the avalanche shapes and induces also a dependence of the asymmetry parameter $a$ on $Tk^{\Delta_k}$ (via the large positive skewness visible in Supplementary Figure S4). In the mean field infinite range model, the cutoff-induced distortion is a symmetric "flattening" of the average avalanche shape, and the asymmetry parameter $a$ is always zero [16,20].

**Supplementary Note 6: Computing the skewness of the average avalanche shape.** The skewness of the avalanches is computed by interpreting their average shapes as probability density functions of $t/T$ in the interval $t/T \in [0,1]$, $P(t/T) \propto [t/T(1-t/T)]^{\gamma-1}[1-a(t/T-1/2)]$ [15]. Thus, the skewness, usually denoted by $\gamma_1$, is given by

$$\gamma_1 = E\left[\left(\frac{t/T-\mu}{\sigma}\right)^3\right], \tag{S8}$$

where $E(\dots)$ is the expectation value, and $\mu$ and $\sigma$ are the expectation value and the standard deviation of $t/T$, respectively. An exact expression for the skewness for a given $\gamma$ as a function of the asymmetry parameter $a$ can be computed, but it is a complicated function, and thus gives little insight as such. Therefore, we report in Supplementary Figure S7 the numerically evaluated skewness as a function of $a$ for the three different $\gamma$-values observable in the interface depinning model by varying $\alpha$. For $a$ close to 0, there is a roughly linear proportionality between the skewness and $a$. In Supplementary Figure S7 we also show three points corresponding to the pairs of skewness and $a$-values measured from the average avalanche shapes of the interface depinning model for different $\alpha$. An analogous relation between $b$ in equation (S3) can be obtained, again exhibiting a linear proportionality between $b$ and the avalanche skewness for small $b$ (not shown).

**Supplementary Discussion:** We have established a scaling picture elegantly summarizing the dependence of the average avalanche shape on the universality class of the avalanche dynamics. From a theoretical point of view, this raises several questions worth future studies: While in the mean-field limit the avalanche shape and other observables can be found exactly within the Alessandro-Beatrice-Bertotti-Montorsi model, can the leading order scaling form for the avalanche shapes we obtain from simple scaling arguments, as well as the small temporal asymmetry we observe numerically, be derived from a theory formulated around the upper critical dimension in terms of an $\epsilon$-expansion [20,22,40]? Is the small temporal asymmetry of the avalanches we observe connected to some properties of other observables, such as the two-point correlation functions, or the shapes of the scaling functions of, say, the avalanche size distributions? The usual two-point correlation function of (interface) fluctuations would not exhibit any signs of that, but of course correlations of avalanche activity *during an avalanche* will do. Better statistics of similar activity maps [32] from experiments would suffice for that. Another question is how does our scaling picture extend to systems where the universality class is controlled not by the range of the elastic interactions, but e.g. by the presence of non-linear terms in the equation of motion, as e.g. in the quenched Kardar-Parisi-Zhang equation [41]? Experimentally the key questions are related to the feasibility of observing the relatively small temporal asymmetry predicted by the depinning models, e.g. in the case of domain wall dynamics in ferromagnetic thin films [33], or in fluid invasion into disordered media [34]. In general, avalanche shapes should depend on the universality class via the values of the critical exponents, and the inherently time-irreversible character of the avalanche dynamics should be manifested as temporally asymmetric avalanches.

## Supplementary References

[38] Dobrinevski, A. PhD Thesis (2013); Dobrinevski, A., Le Doussal, P. & Wiese, K.J. to be published.

[39] Leschhorn, H. Interface depinning in a disordered medium - numerical results. *Physica A* **195**, 324 (1993).

[40] Kolton, A.B., Schehr, G. & Le Doussal, P. Universal Nonstationary Dynamics at the Depinning Transition. *Phys. Rev. Lett.* **103**, 160602 (2009).

[41] Chen, Y.-J., Papanikolaou, S., Sethna, J. P., Zapperi, S. & Durin, G. Avalanche spatial structure and multivariable scaling functions: Sizes, heights, widths, and views through windows. *Phys. Rev. E* **84**, 061103 (2011).
